# Effective Focal Laser Photocoagulation for Persistent Central Serous Chorioretinopathy: A Forgotten Technique

**DOI:** 10.1055/a-2227-4037

**Published:** 2024-02-14

**Authors:** Peter Kiraly

**Affiliations:** 1 Oxford Eye Hospital, Oxford University Hospitals NHS Foundation Trust, Oxford, United Kingdom of Great Britain and Northern Ireland; 2 Nuffield Laboratory of Ophthalmology, University of Oxford Nuffield Laboratory of Ophthalmology, Oxford, United Kingdom of Great Britain and Northern Ireland

## Introduction

Central serous chorioretinopathy (CSC) ranks as the fourth most prevalent nonsurgical maculopathy. It is characterized by a thickened choroid, pigment epithelium (RPE) irregularities, and accumulation of subretinal fluid (SRF) 1 , 2 . In acute CSC cases, visual impairment is typically confined to the area of SRF accumulation and improves once the SRF resolves 3 . In chronic CSC, where SRF fluids persists for at least 6 months, vision loss can become irreversible, potentially leading to severe visual impairment or even legal blindness 4 . Currently, there is no consensus on the duration of SRF persistence required to cause irreversible vision loss. The first treatment modality described for CSC was focal laser photocoagulation (FLP), targeting the site of focal leakage observed during fluorescein angiography (FA) 5 , 6 . FLP involves utilizing a green or yellow laser beam to induce coagulation of tissue at and around the leaking area, effectively sealing the site of leakage 5 . Burumcek et al. demonstrated that CSC patients treated with focal FLP experienced faster resorption of SRF and improved visual acuity (VA) compared to those following a natural course 5 . A significant disadvantage of focal FLP is the possibility of treating only extrafoveal areas of leakage 7 . Moreover, scotoma and secondary choroidal neovascularization (CNV) have been described in CSC patients following treatment with FLP 8 . With the emergence of new treatment modalities, such as half-dose/fluence photodynamic therapy (PDT) 9 , subthreshold micropulse laser (SML) treatment 9 , and oral spironolactone/eplerenone treatment 10 , FLP has largely fallen out of use. Large multicenter trials have compared half-dose PDT, SML, and mineralocorticoid receptor antagonists against each other 9 , 11 , demonstrating the superiority of PDT. Moreover, a study showed that eplerenone was not superior over a placebo in chronic CSC following a 12-month treatment period 12 . However, FLP has not been directly compared to alternative treatment modalities in big multicenter prospective trials. Although half-dose/fluence PDT is unequivocally the most effective treatment modality for chronic CSC, it is associated with high costs and limited availability due to a worldwide shortage of verteporfin 13 , 14 . In the absence of any other effective treatment and with limited PDT availability, patients are often merely observed, experiencing fluctuating SRF and a steady, yet progressively irreversible, worsening of vision. In our case report, we present a patient with persistent CSC and extrafoveal pigment epithelial detachment (PED), who was on the waiting list to receive half-dose PDT treatment. Due to the extended waiting time, he opted for FLP treatment, which resulted in complete resolution of SRF and vision improvement within 1 month after the treatment.

## Case Report

**Fig. 1:**
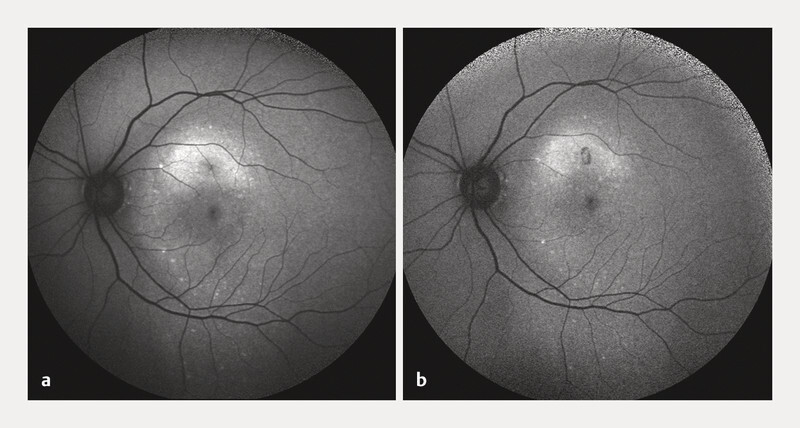
Fundus autofluorescence images before and after focal laser photocoagulation (FLP) treatment. **a** FAF prior to treatment shows hyperautofluorescence superior to the fovea, tracking downwards and resembling an early gravitational track. An area of hypoautofluorescence around the fovea corresponds to the subretinal fluid. **b** FAF captured 1 month post-FLP treatment reveals the resolution of both the subretinal fluid and hypoautofluorescence, with an enlarged area of hypoautofluorescence at the site of the FLP treatment.

**Fig. 2:**
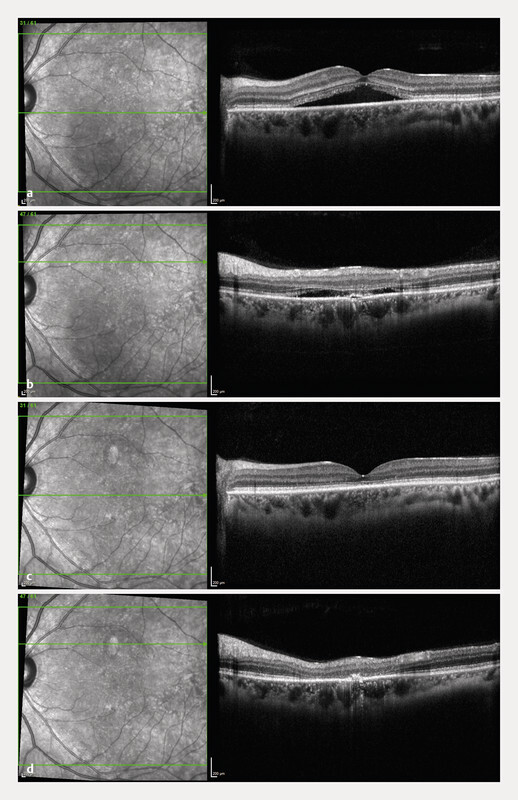
Optical coherence tomography (OCT) showing subretinal fluid (SRF) accumulation and disruption of the outer retinal layers under the fovea ( **a** ), and perifoveal superior pigment epithelium detachment (PED) just before focal laser photocoagulation ( **b** ). OCT images taken 1 month after the treatment show complete SRF reabsorption under the fovea ( **c** ) and around the leaking PED ( **d** ).

A 33-year-old man presented with scotoma and metamorphopsia in the left eye, persisting for a few days. The patient has experienced 4 episodes of CSC, each resolving spontaneously within 2 – 3 months. He denied any use of exogenous steroids; however, he reported experiencing stress at work and at home due to a newborn baby. Best-corrected visual acuity (BCVA) in the right eye was 6/6, and 6/9 in the left eye; intraocular pressure was within normal limits. The slit lamp examination of the right eye was unremarkable. In the left eye, macular edema and pigmentary changes were observed. Optical coherence tomography (OCT) in the right eye revealed pachyvessels with normal choroidal thickness, with no RPE changes and/or SRF accumulation. OCT in the left eye revealed SRF accumulation under the fovea and superior to it, with small PED extrafoveally superiorly with subretinal hyperreflective material around the PED. Moreover, a thickened choroid with pachyvessels and subretinal and intraretinal hyperreflective foci were noted. Fundus autofluorescence (FAF) of the right eye was unremarkable. In the left eye, hyperautofluorescence was seen superior to the fovea, tracking downwards and resembling an early gravitational track ( Fig. 1 a ). After 3 months of persistent and increasing SRF volume ( Fig. 2 a, b ), BCVA in the left eye dropped to 6/12, and the patient was listed for half-dose PDT in the left eye. However, due to a lack of availability of verteporfin, the patient did not receive the treatment for the next 6 months. Meanwhile, the BCVA in the left eye dropped to 6/30, causing the patient to become very upset due to the worsening of visual function. Consequently, he decided to proceed with FLP. The FLP treatment was conducted using a 577 nm laser (Supra Scan 577; Quantel Medical, Cournon dʼAuvergne, France), applying 3 laser spots on and around the area of the leaking PED (300 mW, 200 µm, 0.1 s) to obtain a greyish response on the retina. One month after the FLP treatment, complete SRF resolution was obtained ( Fig. 2 c, d ), along with significant subjective vision improvement and an objective BCVA improvement to 6/12. On FAF, hypoautofluorescence at the fovea resolved with the resolution of SRF, and an enlarged area of hypoautofluorescence was observed at the site of the FLP treatment ( Fig. 1 b ). Fundus examination in the left eye revealed a tiny area of retinal atrophy where FLP was performed. The patient reported no visual field defects.

## Discussion

While FLP is not a novel approach and was the initial treatment method for CSC, its efficacy, simplicity, patient friendliness, and good safety profile are often overlooked by clinicians. In our presented case, we successfully treated the patient with FLP in one session with no need for angiography. Our patient experienced 4 previous episodes of CSC, all of which resolved spontaneously within 2 – 3 months. Despite quick spontaneous resolution of SRF, RPE changes observed on FAF were most likely associated with previous CSC episodes ( Fig. 1 a ). Given the history of spontaneous resolution within 2 – 3 months, we initially opted for observation only during the first 3 months. However, in this instance, SRF under the fovea persisted for 9 months and was accompanied by significant objective and subjective vision loss. Therefore, the patient was very keen to proceed with treatment. Regarding its mechanism of action, FLP acts to seal the area that leaks, which is most commonly associated with RPE irregularities and leaking PED 15 . Experimental studies indicate that adjacent RPE cells expand to cover the gaps created by laser treatment, restoring the RPE barrier 16 . When comparing a cohort of CSC patients after FLP treatment to those following the natural course of CSC, the authors observed a more rapid resolution 5 , 17 , 18 and a lower likelihood of recurrence in the treated patients 19 . Only two small prospective studies have compared FLP and PDT in patients with chronic CSC. These studies showed faster SRF resolution in the PDT cohort but similar long-term morphological and functional outcomes 20 , 21 . Both studies involved patients with chronic CSC and diffuse leakage, in which FLP may be less effective than PDT 20 , 21 . We believe that since FLP targets the RPE, ideal candidates for effective FLP treatment should have only focal leakage that is at least one disc diameter away from the fovea. On the other hand, because PDT targets choroidal hyperpermeability, it is effective for both focal and diffuse leakage, and can be applied irrespective of whether the leakage is foveal or extrafoveal 7 , 9 , 11 . Thus, only future studies that include CSC patients with extrafoveal focal leakage will ascertain the efficacy of FLP compared to the natural course or PDT. In our patient, FLP was performed in less than 5 minutes, with 3 laser spots applied on and around the area of the PED, without FA or indocyanine green angiography (ICGA) prior to treatment. Several clues suggested that the treated PED was the source of SRF leakage beneath the neurosensory retina without confirmation with angiography. In CSC, leakage into the subretinal space is typically associated with RPE irregularities and PED, and given our patient had just one PED, it was likely the leakage source. Additionally, the anatomical location of the PED at the upper margin of the neurosensory detachment suggests that gravitational force contributed to the inferior accumulation of SRF. Furthermore, our patient had a pachyvessel beneath the PED and subretinal hyperreflective material surrounding the PED, both of which are also linked to the area of leakage in CSC 1 , 22 , 23 . Therefore, considering all indirect indicators of leakage, some CSC patients may not require angiography before FLP treatment, streamlining the treatment planning process. In our case, due to previously confirmed CSC episodes, FA and ICGA were not utilized for establishing the diagnosis and treatment planning. Nevertheless, FA and ICGA continue to be important imaging modalities to establish the diagnosis of CSC. In terms of the safety profile, prior studies have reported scotoma and secondary CNV in CSC patients following FLP treatment 1 , 7 , 15 , 24 . Scotoma after FLP develops due to laser photocoagulation of the PED, which leads to focal atrophy of the outer retinal layers ( Fig. 2 d ). A natural history study of PED in CSC indicated that the majority of PEDs spontaneously resolve, with subsequent RPE atrophy developing in 86% of previous PEDs 25 . Hence, CSC patients are likely to develop a scotoma related to outer retinal layer atrophy, whether or not they undergo FLP treatment. Iatrogenic CNV following FLP in CSC is very rare, being reported in only 11 of 1824 treated eyes (0.60%) 26 . Most reports of iatrogenic CNV following FLP in CSC date back a few decades, when treatments were administered using older generations of laser machines, which may have been more susceptible to inducing iatrogenic CNV. Furthermore, those reports did not utilize newer multimodal imaging techniques like OCT and OCT angiography, which can detect subtle CNV before FLP 27 . At the time, it was not widely recognized that secondary CNV is associated with chronic CSC in 24% of cases, as documented by Mrejen et al. 4 . As a result, CNV might have been erroneously attributed to FLP treatment rather than the natural progression in chronic CSC patients. Retinal laser photocoagulation is widely used in everyday medical retina practice, primarily for treating diabetic retinopathy and retinal vein occlusions. Therefore, FLP for selected patients with CSC could potentially be more widely available and cost-effective, as it eliminates the need for drugs required in treatments like PDT. On the other hand, PDT is usually done only in bigger academic centers with a small number of retinal specialists performing it. Moreover, since July 2021, there has been a global shortage of verteporfin, a critical drug used for PDT 14 . Considering global challenges with verteporfin shortages, limited access to PDT-trained centers, and suboptimal outcomes with other modalities, FLP emerges as a promising option for CSC patients with extrafoveal leakage. Unfortunately, CSC patients with extrafoveal leakage, who could be effectively treated with FLP, are frequently just observed by retinal clinicians, with fluctuating SRF and steady but progressive irreversible vision worsening. Despite focal laser photocoagulationʼs historical presence in CSC treatment, our case serves as a pertinent reminder for retinal specialists to reconsider this treatment approach.
